# Skill-learning by observation-training with patients after traumatic brain injury

**DOI:** 10.3389/fnhum.2022.940075

**Published:** 2022-08-31

**Authors:** Einat Avraham, Yaron Sacher, Rinatia Maaravi-Hesseg, Avi Karni, Ravid Doron

**Affiliations:** ^1^School of Behavioral Science, The Academic College, Tel Aviv-Yafo, Israel; ^2^The Loewenstein Rehabilitation Medical Center, Ra’anana, Israel; ^3^Sackler Medical Faculty, Tel Aviv University, Tel Aviv-Yafo, Israel; ^4^Sagol Department of Neurobiology, Faculty of Natural Sciences, Brain–Behavior Research Center, University of Haifa, Haifa, Israel; ^5^Sheba Medical Center, Ramat Gan, Israel; ^6^Department of Education and Psychology, The Open University, Ra’anana, Israel

**Keywords:** skill acquisition, traumatic brain injury, mirror neurons, procedural memory, action observation, rehabilitation after brain injury, motor performance

## Abstract

Traumatic brain injury (TBI) is a major cause of death and disability in Western society, and often results in functional and neuropsychological abnormalities. Memory impairment is one of the most significant cognitive implications after TBI. In the current study we investigated procedural memory acquisition by observational training in TBI patients. It was previously found that while practicing a new motor skill, patients engage in all three phases of skill learning–fast acquisition, between-session consolidation, and long-term retention, though their pattern of learning is atypical compared to healthy participants. A different set of studies showed that training by observing a motor task, generally prompted effective acquisition and consolidation of procedural knowledge in healthy participants. The aim of our study was to (i) evaluate the potential benefit of action observation in TBI patients. (ii) Examine the possibility of general improvement in performance between the first (24 h post-training) and second (2 weeks post-training) stage of the study. (iii) Investigate the link between patients’ ability to benefit from observational learning (*via* performance gains–speed and accuracy) and common measures of injury (such as severity of injury, functional and cognitive measures).

**Materials and methods:**

Patients hospitalized after moderate to severe TBI, were trained by observation for the finger opposition sequence (FOS) motor task. They were then tested for the observation-trained sequence (A) and a similar control sequence (B), at two different time-points (24 h post-training and 2 weeks later).

**Results revealed:**

(i) a significant difference in performance between the trained (A) and untrained (B) sequences, in favor of the trained sequence. (ii) An increase in performance for both sequences A and B toward the second (retention) session. (iii) The advantage for sequence A was stable and preserved also in the second session. (iv) Participants with lower moderate Functional Independence Measure (FIM) scores gained more from observational-procedural learning, compared with patients with higher functional abilities.

**Conclusion:**

Overall, these findings support the notion that TBI patients may achieve procedural memory consolidation and retention through observational learning. Moreover, different functional traits may predict the outcomes of observational training in different patients. These findings may have significant practical implications in the future, regarding skill acquisition methods in TBI patients.

## Introduction

Traumatic brain injury (TBI) is a disruption of brain function and structure due to the application of an external physical force. TBI is a major cause of death and disability in Western society. TBIs can cause neuroanatomical abnormalities including brain contusions, cerebral edema, ischemia, and hemorrhages (Bigler et al., 1989), though one of the most common outcomes of head trauma is widespread diffuse axonal injury (DAI). This type of damage is considered the least focal and is difficult to detect, though brain imaging suggests that the most vulnerable cortical areas in DAIs are the mesial temporal and lateral frontal lobes (Bigler, 2015; Maxwell, 2015); Ventricular enlargement was also observed (Crosson et al., 1993).

Traumatic brain injury has many cognitive implications that prevent patients from fully recovering functionally. Memory impairment is one of the most significant consequences (Levin, 1989; Vakil, 2005) and has been widely investigated in patients who have sustained TBI (Vakil, 2005).

### Memory and skill learning

Over the past three decades, numerous studies have demonstrated that memory is a complex construct (Squire, 2004), composed of different levels of codependent structures and mechanisms, involving both explicit and implicit domains (Vakil, 2005). Procedural learning is considered an implicit type of learning, engaging the procedural (”*how to*”) memory system, designed for skilled performance gained through multiple repetitions (Adams, 1987).

Procedural learning is obtained through several distinct phases. The early phase of “fast learning”, requires a critical but limited amount of task repetitions, which causes significant improvement in performance. The next is a plateau phase, in which further practice is not likely to improve performance (Hauptmann and Karni, 2002; Korman et al., 2003). The last is the “slow learning” phase, in which robust delayed (”off-line”) gains can be attained between and across several training sessions, without any additional practice (Karni et al., 1994, 1998; Walker, 2005). This last phase reflects a latent memory consolidation process, triggered by training but continually evolving hours after training has ceased (Karni et al., 1995; Ari-Even Roth et al., 2005; Walker, 2005; Dorfberger et al., 2007), and for some tasks requires sleep in order to complete (Doyon et al., 2009; Debas et al., 2010). The skill may then be retained for months and even years (Karni, 1996).

Extensive research has been conducted to investigate the effects of TBI on memory processes, although the main focus in those studies is most commonly placed on explicit memory processes rather than implicit process such as skill learning (Vakil, 2005), perhaps due to the fact that even severe TBI usually results in minimal losses of skills acquired prior to the injury (Schmitter-Edgecombe and Beglinger, 2001; Vakil et al., 2002). Nevertheless, it remains unclear whether these patients’ ability to acquire new skills remains intact following TBI.

**FIGURE 1:**
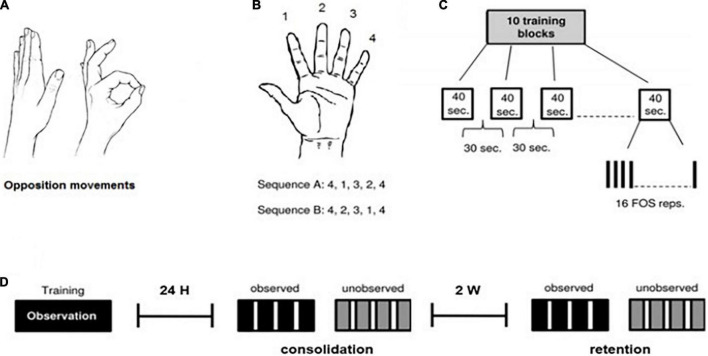
The finger-to-thumb opposition task (FOS) and training protocol. All participants were trained and tested on the finger-to-thumb opposition sequence (FOS) learning task (Korman et al., 2003). **(A,B)** Two five-element sequences, each the reverse of the other, were used with each participant. Participants were instructed to oppose the fingers of the trained hand to the thumb in a given five-element sequence, as fast and accurately as possible. **(C)** The training protocol included 160 repetitions of the assigned FOS (10 training-blocks of 16 repetitions) as in Korman et al. (2003). **(D)** Timelines of the experiment procedure: Each participant was trained, observing sequence A (training protocol as presented above), and was tested 24 h post-training (consolidation/overnight test) and 2 weeks post-training (retention test) for both sequence A (observed) and B (unobserved). Each test session was composed of 4 blocks, 30 s each, with a 50 s interval between testing-blocks.

To explore this question, Korman et al. (2018) conducted a clinical study measuring the ability of moderate-severe TBI patients to learn a new skill, compared with healthy participants. In this study, sub-acute patients hospitalized for rehabilitation, and healthy participants, were trained for several consecutive days, using the finger-to-thumb opposition sequence (FOS) learning task (see Figure 1). Participants were then tested for performance gains at different time-points during the training protocol. The patients’ baseline performances were slower compared with healthy participants, and they demonstrated within-sessions losses in performance, which was suggested to reflect cognitive fatigue. Despite these differences, the overall results revealed effective memory consolidation processes and long-term retention in patients with TBI, similar to the healthy participants.

### Motor learning by action observation

Over the past decade, several studies have suggested that the observation of an action can lead to specific subsequent performance gains (Heyes and Foster, 2002; Vinter and Perruchet, 2002; Torriero et al., 2007; Van Der Werf et al., 2009; Hayes et al., 2010; Zhang et al., 2011), as it may provide a “perceptual blueprint” for the observer (Doody et al., 1985) and therefore facilitate procedural learning processes. In terms of observational skill learning, there is evidence that even new motor tasks, for which no representation is available in the observer’s repertoire, can be acquired (Torriero et al., 2007; Cross et al., 2009; Boutin et al., 2010).

Motor learning by observation is suggested to involve a specific neurological system, called the “mirror neurons” system. Mirror neurons are a particular class of visual-motor neuron circuitry, first discovered in the macaque monkey, though evidence from functional neuroimaging studies contributes to the idea of a homologous “mirror neuron system” in humans, with the lower part of the pre-central gyrus, plus the posterior inferior frontal gyrus (pIFG) and anterior inferior parietal lobule (aIPL), constituting core regions of the system (Rizzolatti et al., 1996; Buccino et al., 2001).

Some evidence-based studies over the past decades (e.g., Calvo-Merino et al., 2006; Cross et al., 2009) identified a broader set of brain regions that are active both when observing and performing actions. These regions are referred to as the “action observation network” (AON), a network of neural regions involved in visual analysis of action as well as areas involved in visuomotor and sequence learning (e.g., Jenkins et al., 1994; Sakai et al., 2002; Grèzes et al., 2003).

A recent set of studies, performed with healthy participants (Maaravi-Hesseg, 2006; Hesseg et al., 2016; Hesseg, 2017), showed that training by observation was an effective practice technique not only for the acquisition, but also for the consolidation and long-term retention of the trained FOS task. These studies investigated whether procedural knowledge can be acquired from or augmented by training methods that do not involve actual physical performance of the task-related movements. Their findings demonstrated not only that a combined strategy, involving both observation and training, was an effective means for learning, but also that observation alone is an effective learning technique (also Gatti et al., 2013).

Several questions remain regarding patients with TBI: is skill acquisition by observation a plausible and effective learning method? Is the AON expected to engage similarly in TBI patients, considering the significant changes in neuro-functioning and anatomy previously discussed? Neuroimaging studies of stroke patients have demonstrated significantly higher activation of motor areas after action observation training interventions, as well as long term behavioral improvements (Ertelt et al., 2007; Garrison et al., 2013; Brunner et al., 2014), even in patients in the chronic phase of disability (Ertelt et al., 2007). Garrison et al. (2013) showed that action observation may even activate the lesioned hemisphere in an effector-specific manner.

### The current study

The aim of this study is (i) to investigate whether people who suffered a moderate-severe TBI may benefit from action observation in acquiring a new motor skill; (ii) to examine the possibility of improvement in performance between different time-points during the study; and (iii) to investigate correlations between different measures of performance with common measures of injury and demographic measures (e.g., age, severity of injury, functional and cognitive measures).

The protocol used as the basis for the observation training session, is the standard FOS (finger opposition sequence) task developed by Korman et al. (2003) (see Figure 1), through observation only, while comparing participants’ performance in a trained (observed) sequence, with a new (unobserved, control) sequence. This task has been repeatedly shown not only to improve performance across the training session but also to trigger post-training consolidation phase gains as well as effective retention of the skill (Korman et al., 2003, 2007; Dorfberger et al., 2007; Adi-japha and Karni, 2016).

## Materials and methods

### Participants

Research included eleven moderate-severe TBI patients (all males, ages 18 to 65), who were hospitalized for rehabilitation in the sub-acute phase and met inclusion criteria for the study. Participants were tested while hospitalized through all phases of the study, all suffered moderate to severe TBI at least 2 months prior to the beginning of the study. 10 participants suffered a non-penetrating brain injury, and one participant had a penetrating shrapnel injury. Patients with clinical depression, anxiety or previous neurological or psychiatric disease were excluded from the study. Also excluded were patients who suffered a direct orthopedic or neurological trauma to the upper limb to be trained, or severe pain that might limit wrist and finger movement. Participants’ ability to follow task instructions and perform all 4 finger-opposition movements with the to-be-trained hand was assessed. Patients with previous expertise on finger movement sequences (e.g., musicians and professional typists) were also excluded from the study. Among patients included in the study, one failed to complete the final session of the study (retention test, 2 weeks after training), due to an early discharge from the hospital.

All patients performed the sequence with their best-functioning limb: eight patients used their right dominant hand, one used his left dominant hand, and two used their left non-dominant hand.

Table 1 summarizes all details regarding patients’ characteristics relevant to this study.

**TABLE 1:** Patient’s characteristics.

Serial no.	Age	Time from injury (weeks)	Gad7 scale (1–21)	Beck scale (0–63)	GCS (1–15)	FIM (18–126)		Fugel Meyer (1–60)	RBMT (1–24)	Imaging finding	Physical and behavioral findings	Dominant hand	Trained hand
												
						In admission	In entering the study						
**1**	22	15	2	12	3	60	119	60	20	RT PF hemorrhage, Sharpnels	Executive dysfunctions	Left	Left
**2**	24	16	5	15	3	94	96	60	19	Bilateral PF contusion	Executive dysfunctions	Right	Right
**3**	21	15	1	0	3	73	92	60	16	Rt FT contusion, Lt Par contusion	Memory and executive dysfunctions	Right	Right
**4**	22	9	1	1	3	117	117	60	NA	Lt frontal contusion	Executive dysfunctions	Right	right
**5**	35	14	4	15	3	71	108	60	11	DAI	Memory and executive dysfunctions	Right	Right
**6**	29	12	1	1	3	72	92	52	14	Lt subdural and sub arachnoid hemorrhage	Rt hemiparesis, Executive dysfunctions	Right	Left
**7**	38	32	1	1	3	21	96	56	19	Rt FP hypodense areas	Memory and executive dysfunctions	Right	Right
**8**	21	26	0	3	4	30	61	60	NA	Lt subdural hematoma, hydrocephalus, vp shunt		Right	Left
**9**	61	24	1	1	3	39	60	56	8	DAI	Double hemiparesis, memory and Executive dysfunctions	Right	Right
**10**	65	10	5	3	15 on admission, deteriorated to 7	97	107	59	20	Pneumocephalus on early CT, bilateral acute SDH on follow-up study	Lt Hemiparesis and hypoesthesia, Executive dysfunctions	Right	Right
**11**	61	10	3	8	3	79	120	55	16	Left temporal, bilateral dorsal parietal and right frontal subarachnoid hemorrhage	Executive dysfunctions	Right	Right
**Mean**	36.27	16.63	2.18	5.45	4.18	68.45	97.09	58	15.88				
**Std.**	17.69	7.47	1.77	5.93	3.6	29.4	20.85	2.79	4.22				

### Measures

Measures of exclusion criteria included the Fugl–Meyer test (Feys et al., 2000) for upper-limb motor assessment, and the visual analog scale (VAS) for pain assessment (Bijur et al., 2001). Severity of injury was assessed by the Glasgow Coma Scale (GCS) (Teasdale and Jennett, 1974) and through anamnestic information regarding the duration of loss of consciousness and post-traumatic amnesia. Brain imaging findings were drawn from medical records. The Beck Depression Inventory Scale (BDI, ranges from 0 to 63) and the General Anxiety scale (GAD7, ranges from 0 to 21) were used for emotional screening. Scores higher than 16 points were the exclusion criterion for depression (Beck et al., 1988) and scores higher than 7 points were the exclusion criterion for anxiety (Plummer et al., 2015).

Functional evaluation was assessed with the Functional Independence Measure (FIM) (Seel et al., 2007) measuring activities of daily living (ADL) abilities. Levels of explicit memory impairments were assessed by the Rivermead Behavioral Memory Test (RBMT) battery for memory evaluation (Wiseman et al., 2000).

All data and patients’ characteristics (presented in Table 1) was drawn from the medical records, with the exception of the emotional screening and motor assessment, evaluated separately before the beginning of the study.

### Task and procedure

The experiment included 3 sessions: a training session and two subsequent test sessions, at 24 h after training, and 2 weeks later (see Figure 1). All subjects participated in a single-session observation-training protocol for a given sequence of finger opposition movements (sequence A, observed sequence). 24 h after the observation-training session, participants were tested for both the trained sequence A (the consolidation test) and for an unobserved control sequence B. An identical test session was conducted 2 weeks later (the retention test).

During the training session, subjects were shown and explicitly instructed on a 5-element sequence of finger to thumb opposition movements (FOS) numbering the fingers 1–4, with 1 designating the index finger and 4 the little finger: 4-1-3-2-4 (Sequence A). Once a participant was able to repeat the sequence accurately for three consecutive times, indicating that he knew what movements were required to execute the sequence correctly, a video of a hand was shown (matching his pre-determined trained hand), illustrating a direct (palm facing) view of Sequence A. The video was composed of 10 blocks of 16 repetitions of the sequence, each sequence cued at a comfortable rate. Thus, the practice session included altogether 160 repetitions of the FOS. To ensure that the training session is mostly an observatory session, training was designed to involve minimal physical hand movement, and participants were asked to place both hands on the table while watching the video. At two different points during the observation-training, participants were asked to perform “catch trials”–a single demonstration of the sequence presented in the video, in order to make sure they were attentive to the sequence presented. After watching the video, participants were instructed to return to their daily routine at the hospital (physiotherapy, occupational therapy etc.) and avoid further engagement with the task.

Twenty-four hours after the training session ended, participants were tested on their performance of the trained sequence (A) and a control sequence of equal length and complexity, composed of the reverse combination of the same elements: 4-2-3-1-4 (Sequence B). The observed sequence A, and the unobserved sequence B, were composed of identical movements and number of movements per digit, and differed only in the order of their components (Hesseg, 2017).

The control sequence was presented to the subjects right after they were tested for sequence A. The control sequence was presented to the participants similarly to sequence A, and accordingly, the test for sequence B was performed after participants were able to execute the sequence correctly three consecutive times.

The performance tests consisted of four intervals (blocks) of 30 s each, followed by a 50 s break, with clear auditory “start” and “stop” cues. The participants performed the instructed movements in direct view of a video camera, to allow recording of all finger movements. During the test for both sequences, participants were encouraged to perform the sequence “as quickly and accurately as possible” and were instructed to divert their gaze from their hand so that visual feedback was not afforded. Participants were instructed that if they become aware of committing an error they should carry on and resume the next sequence.

An additional identical test session (retention test) was conducted 2 weeks after the consolidation test was completed. No further training was performed during the weeks between the consolidation and retention test.

### Statistical analysis

Statistical tests were run using Statistical Package for the Social Sciences (SPSS Statistics for Windows, Version 26; IBM Corp., Armonk, NY, United States).

For each participant, two performance measures were calculated for each test-block: the number of correctly completed sequences, as a measure of speed and the number of sequencing errors committed in each test-block, as a measure of accuracy. The two measures of performance, (speed and accuracy) were analyzed separately after all data was collected. Speed and accuracy were calculated as the average performance across the 4 corresponding test blocks.

To evaluate *the advantage of observational learning* (the difference between sequence A and B), paired *t*-test comparisons were conducted for each performance measure (speed/accuracy) and each session (overnight test/retention test 2 weeks later), comparing the observed (A) and unobserved sequences (B).

To test for *improvement in performance over time* (between test sessions), paired *t*-test comparisons were conducted for each performance measure (speed/accuracy) and for each sequences (A/B), comparing performance between the two test sessions (overnight test/retention test 2 weeks later).

To evaluate *between session gains*, repeated-measures ANOVAs (2 × 2) were performed for each measure separately (speed/accuracy), with test session (24 h/2 weeks) and sequence (A/B) as within-subject factors.

To test for *within session gains* in specific phases (improvement in performance through test blocks), repeated-measures ANOVAs (2 × 2 × 4) were conducted for each performance measure separately (speed/accuracy), with test session (24 h/2 weeks), sequence (A/B) and test-block (1/2/3/4) as within-subject factors. *Post-hoc* analysis for within-session gains was conducted to evaluate interaction effects found in rm-ANOVAs–using a single planned paired comparison (*t*-test), comparing performance speed for sequence A, between the first (1) and second (2) test block in each session (overnight/retention).

Finally, Pearson’s matrix were calculated to assess the correlations between performance (speed and accuracy as well as gains in those parameters) and continues variables relating to patient’s function and demographic measures (FIM in admission to the hospital and on trial entrance, RBMT score, Fugl-Meyer score, GCS, level of severity, time from injury and age).

Figure 1 demonstrates the protocol task and procedure.

*Research was conducted during the months of July–September 2019 at the Loewenstein rehabilitation Medical Center in Ra’anana, Israel. The study was approved by the Human Research Ethics Committee of the Loewenstein Rehabilitation Medical Center and the Israeli Ministry of Health. All participants and their guardians gave written informed consent before inclusion.

## Results

Table 2 summarises participants performance: mean performance (speed and accuracy) and standard deviation for both test sessions (overnight and retention).

**TABLE 2:** Mean performance (speed and accuracy) and standard deviation for both test sessions (overnight and retention).

		Observed sequence (A)		Unobserved sequence (B)		
			
Session	Performance parameter	Mean	Std. deviation	Mean	Std. deviation	*N*
**Overnight test**	Correct seq.	11.204	2.917	8.25	3.092	11
	errors	0.568	1.113	1.795	1.576	11
**Retention test**	Correct seq.	13.4	4.147	10.875	4.222	10
	errors	0.4	0.376	1.6	1.029	10

(i) To evaluate *the advantage of observational learning* (the difference between sequence A and B) in different time-points, paired t-test comparisons were conducted for each performance measure (speed/accuracy) and each session (overnight test/retention test 2 weeks later), comparing the observed (A) and unobserved sequences (B). In the *t*-test, both speed and accuracy were calculated as the average performance across the 4 (corresponding) Test blocks.

**FIGURE 2:**
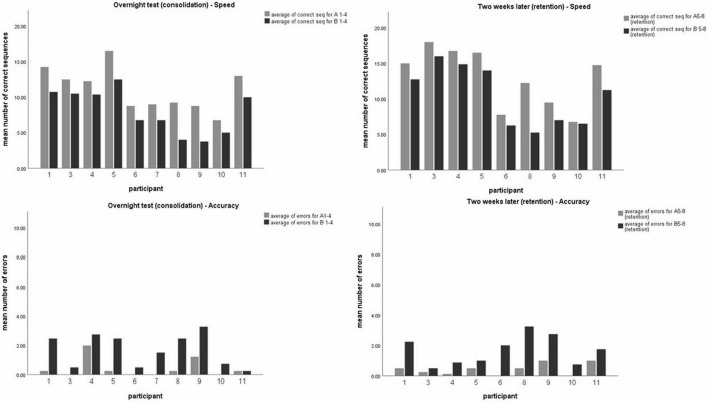
Participants mean level of performance. Mean number of correct sequences (top) and mean number of errors (bottom) of both timelines–24 h post-training (overnight test-left, sequences A1-4 or B1-4) and 2 weeks later (right, sequences A5-8 or B5-8). Each data coulomb represents the mean performance of 4 test-blocks for each participant, for the observed sequence A (gray) and unobserved sequence B (black). Data is presented as mean ± SEM. *Indicates *p* < 0.05, **indicates *p* < 0.001.

**FIGURE 3:**
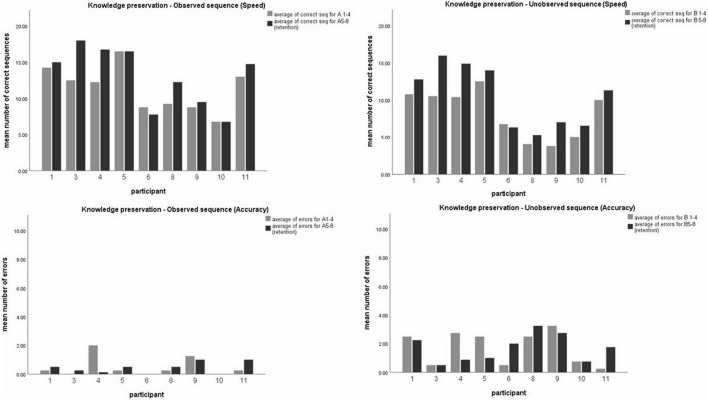
Comparing participants’ performance in the observed and unobserved sequence, 24 h post-training and 2 weeks post-training. Mean number of correct sequence (top) and mean number of errors (bottom) of both the observed (left) and unobserved (right) sequence. Each data coulomb represents the mean performance of 4 test blocks for each participant, while each color represents either the first session (24 h, gray) or the second session (2 weeks, black).

On average, the participants committed very few errors in both the observed (mean = 0.51) and unobserved (mean = 1.71) sequences. Twenty-four hours post-training, a significant difference was shown in performance between the observed (A) and unobserved (B) sequences in both speed (*t* (10) = 7.20, *p* < 0.001) and accuracy (*t* (10) = −4.5, *p* < 0.001) indicating effective learning of sequence A *via* observation. Moreover, a significant difference between sequence A and B was found 2 weeks post-training, in both speed (*t* (9) = 3.80, *p* < 0.05) and accuracy (*t* (9) = −4.31, *p* < 0.05), indicating that the advantage achieved by the observation-training session was preserved (see Figure 2).

(ii) To evaluate whether skills obtained in training sessions were preserved and retained 2 weeks later, intra-subject comparison was made for performance in both sequences A and B. Analysis using *t*-test for paired comparisons revealed a significant increase in performance between the overnight, and 2-weeks post-training test session for both sequences, in speed (*t* (9) = −2.68, *p* < 0.05; *t* (9) = −4.15, *p* < 0.05; for seq A and seq B, respectively) but not in accuracy (*t* (9) = 0.6, *p* > 0.05; *t* (9) = 0.48, *p* > 0.5; for seq A and seq B, respectively), thus demonstrating a general improvement in performance gains 2 weeks later, without a significant change in accuracy (see Figure 3).

**FIGURE 4:**
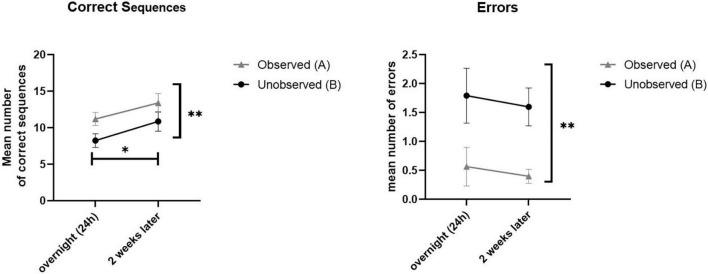
Demonstrated overall performance of observed sequence A (gray), compared to unobserved sequence B (black). Mean performance in speed (correct sequences, left) and levels of accuracy (mean number of errors, right) for both the overnight (24 h) and retention (2 weeks later) sessions.

In analyzing performance, repeated measures ANOVA were calculates separately for speed and accuracy as dependent measures (in a 2 × 2 model, with session and sequence as within-subjects factors), and revealed a significant effect of sequence [*F*_(9)_ = 28.48, *p* < 0.001, η^2^ = 0.76] as well as session [*F*_(9)_ = 12.35, *p* < 0.05, η^2^ = 0.578]. Thus, in terms of speed, performance was significantly higher in trained sequence A compared to the untrained sequence B, across test sessions. Moreover, participants performed better (in speed) in the second test session (at 2 weeks) compared to the first session (at 24 h), for both sequences A and B. In terms of accuracy, the same effect was found significant for sequence [*F*_(9)_ = 21.92, *p* < 0.001, η^2^ = 0.709], but not for test session [*F*_(9)_ = 0.32, *p* > 0.05, η^2^ = 0.034]. In other words, participants had better accuracy (less errors) in the trained sequence A (compared with B) across test sessions, but did not improve in accuracy between the two test sessions, either in sequences A nor B. No session*sequence interaction effects were found for speed [F_(9)_ = 1.26, *p* > 0.05, η^2^ = 0.123] nor for accuracy [*F*_(9)_ = 0.00, *p* > 0.05, η^2^ = 0.000]. Figure 4 demonstrated the main effects found.

**FIGURE 5:**
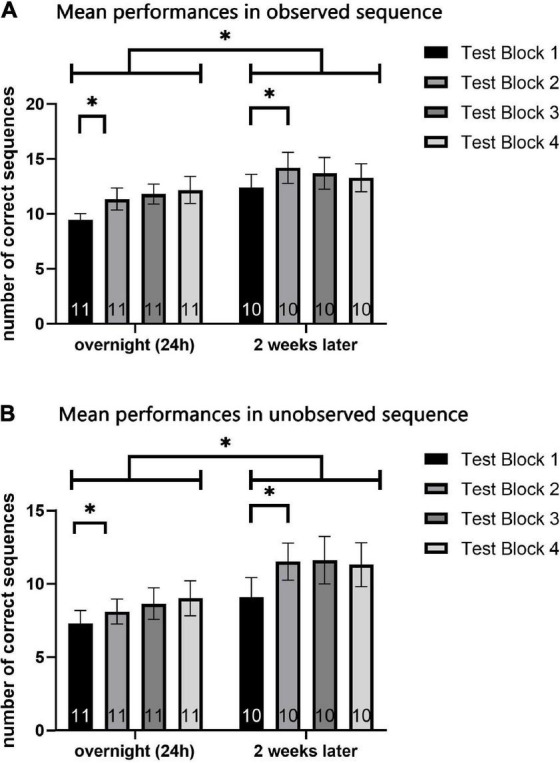
Mean performance in each test-block (1–4) of the overnight test-session and 2-week test-session. Mean number of correct sequences of both the observed **(A)** and unobserved **(B)** sequence. Each data coulomb represents the mean performance of all participants in a specific test-block, while each color represents a different test-block (1 to 4). Data is presented as mean ± SEM. *Indicates *p* < 0.05, **indicates *p* < 0.001.

To evaluate the possible effect of testing sessions on participants’ performance, repeated measures general linear model (GLM) analyses were used to compare the performance between the two sequences (observed A, unobserved B), in the four test-blocks (1–4), of the two testing-sessions (overnight, 2 weeks later), in a within subject 2 × 4 × 2 factor model. The two measures of performance, the number of correct sequences performed within each test-block (speed) and the number of sequencing errors committed in each test-block (accuracy) were analyzed separately (Figure 5 demonstrates results for speed measure).

For both sequences A and B, measurement of the number of correct sequences (the speed performance parameter) revealed a significant effect of training session [*F*_(9)_ = 7.16, *p* < 0.05, η^2^ = 0.443; *F*_(9)_ = 17.2, p = 0.002, η^2^ = 0.656; respectively for sequence A and B] and testing-block [*F*_(7)_ = 7.56, *p* < 0.001, η^2^ = 0.456; *F*_(7)_ = 4.58, *p* < 0.05, η^2^ = 0.337; respectively for sequence A and B]. No major effects of session or of test block, and no interaction effects, were found when analyzing the mean number of errors (the accuracy performance parameter) for sequences A and B.

However, when analyzing the mean number of correct sequences (speed), an interaction effect for test-block*session, was found in sequence A [*F*_(7)_ = 3.13, *p* < 0.05, η^2^ = 0.258] but not in sequence B [*F*_(7)_ = 0.959, *p* > 0.05, η^2^ = 0.096]. *Post-hoc* analyses were performed, to detect the source of interaction in the observed sequence A. In observing the graphs and the descriptive statistics, the effect revealed is an increase in performance from the first to the second block, which was followed by stabilization (see Figure 5). No major differences were shown among the second, third, and fourth test blocks. Therefore, a one-tailed *t*-test was used, as a single planned paired-comparison, between the first and second test block. In each of the testing sessions, paired *t*-test comparisons showed a significant increase between the first test-block and the second (*t* = 2.753, *p* < 0.05; *t* = 4.630, *p* < 0.05; respectively for the overnight and retention test) for the observed sequence A.

(iii) Given the small number of participants and some missing data, the ability to identify reliable correlations in our analyses was limited, though some significant correlations were detected.

Independent Pearson correlation tests showed a significant correlation between FIM measured in admission to rehabilitation (FIM1) and total gains of sequence B (speed only) (*R* = 0.649, *p* < 0.05). A significant correlation was also found between FIM measured in the beginning of study (FIM2) and average of correct sequences for B (overnight) (*R* = 0.745, *p* < 0.05), suggesting a connection between measures of ADL functioning and speed performance of the unobserved sequence. No correlations were found between FIM (both at admission and approximately to the beginning of the study) and total gains for A (speed or accuracy) or total errors for B.

Moreover, a negative correlation was found between FIM2 and the average difference between performance in observed and unobserved sequences in retention test, measured by number of correct sequences (*R* = −0.615, *p* < 0.05). Also, FIM in admission for rehabilitation (FIM1) was negatively correlated with all measures of benefit of observational learning–the gap between A and B in number of correct sequences (*R* = −0.647, *p* < 0.05; *R* = −0.672, *p* < 0.05; respectively for overnight and retention test), and the average difference between A and B in number of errors (*R* = −0.625, *p* < 0.05; *R* = −0.732, *p* < 0.05; respectively for overnight and retention test). Thus, participants with higher FIM measures (initially and to some extent on entering the study) demonstrate a smaller benefit from observational training. Correlations were also tested for FIM scores and measures of general improvement from the first to the second session (the difference between the averages of speed performance in the first and second session); no correlations were found for either sequence A or B.

Finally, no significant correlations were found between individual gains in performance across the study period and the score in the explicit memory test (RBMT) as well as scores obtained for upper-limb motor function (Fugl-Meyer). Also, no correlations were found between any measures of performance, and individual characteristics of patients and injury (GCS, level of severity, time from injury and age).

## Discussion

The main goal of the current study was to investigate the potential of observation-based procedural learning in patients with moderate to severe TBI. Significant differences in performance between the trained and untrained sequences were found in both speed and accuracy, 24 h post-training as well as 2 weeks post-training. This conclusion is in line with previous studies demonstrating preserved consolidation processes and long-term retention of skill learning in patients with TBI (Korman et al., 2018). The results also complement studies exhibiting evidence of observational learning in both healthy (e.g., Caspers et al., 2010; Hesseg, 2017) and post-stroke patients (Ertelt et al., 2007; Franceschini et al., 2012; Brunner et al., 2014). These current findings support the notion that TBI patients may achieve procedural memory consolidation and retention by observational learning, perhaps partially overcoming their post-injury neuronal and cognitive restraints.

Our results demonstrated that the knowledge obtained through observing sequence A was preserved, with performance increases in both sequence A and B in the second (retention) session. This pattern of improvement was found in performance speed but not in number of errors, thus, it is safe to presume that no speed-accuracy trade-off had occurred in the retention test (otherwise, number of errors would rise as speed increases).

The single demonstration of the sequence, performed before the test or training sessions, as well as the “catch trials” (during observational training), are considered insufficient to saturate and induce learning (Hesseg, 2017). Conversely, the test session itself (given its physical practice nature) was indeed expected to serve as a training session, and it was shown to facilitate and enhance performance gains, as demonstrated by within-session gains occurring in the first and second test sessions as well as performance gains obtained between testing sessions. Within-session gains were found significant mainly between the first and second test blocks (out of the 4 test blocks per session), followed by a plateau in performance in test blocks 2 to 4. Previous studies have demonstrated a similar pattern in motor training and have suggested that this saturation phase (which may differ among patients) is crucial for the occurrence of delayed, inter-session gains (Karni et al., 1993; Hauptmann et al., 2005). Given that for each patient saturation may occur after a different number of repetitions, training and rehabilitation protocols should be optimized on an individual basis (Hauptmann and Karni, 2002; Hauptmann et al., 2005).

One might expect a different pattern of performance in the retention test session, perhaps presenting a more moderate difference between the observed and unobserved sequences. Previous studies with healthy participants, have in fact found motor training to have a masking effect on the procedural gains attained by observation (Blandin et al., 1999; Hesseg et al., 2016). Nevertheless, gains achieved from training by observation remained the same from the first and second test sessions. It should be mentioned that only a short video of observational training discriminated between the participants’ experience of sequence A and B at the point retention was assessed. The fact that considerable motor training did not eliminate the effect of observational training indicates just how effective this type of learning might be for TBI patients.

In addition, despite the small number of participants in the current study, marginally significant correlations were found between the FIM (both in admission and while entering the study), and total performance gains in sequence B (but not A). The fact that FIM scores did not predict the level of performance in the observed sequence A may indicate that sequence A and B did not engage similar processes of learning. This notion is in agreement with previous studies, which provided strong indications that the overlap between procedural knowledge acquired from actual movement and that acquired by observing movement may be limited, as demonstrated in imaging studies, as well as behavioral experiments (e.g., Hesseg et al., 2016; Korman et al., 2018).

Moreover, participants with lower FIM scores gained more from observational procedural learning, compared to patients with higher functional abilities. This finding remained significant for both speed and accuracy, and in both testing sessions. In congruence to the previous correlation found in total gains, it is possible that observational learning affected patients differently than motor training.

Several works that categorized patients by FIM scores into distinct groups of lower, middle, and upper bands, found middle-band patients to have the greatest functional improvements after motor rehabilitation, while many upper-band patients had little room to gain (Asberg et al., 1991; Oczkowski and Barreca, 1993; Stineman et al., 1998). Our research involved middle to upper-band patients, thus, it is possible that those patients demonstrated a ceiling effect, manifesting in smaller gains in observational learning.

It is also possible that patients hospitalized for rehabilitation with an initially high FIM score, may represent a different group of patients, for whom the major issues in rehabilitation revolve around cognitive deficits rather than motor ones (e.g., the frontal-injury patients who often struggle with executive functions and/or personality changes rather than motor constraints) (Stuss, 2011). Those patients may also have specific difficulties with observational skill-learning, perhaps due to high levels of distractions and attentional difficulties (Stuss et al., 1999). Future research should explore these questions and investigate the different subgroups of TBI patients (different FIM bands, as well as specific injury involving pre-frontal regions).

We acknowledge that our results and conclusions are based on a small sample of participants with high inter-individual differences in demographic and clinical parameters.

Studies with a larger number of participants may enable us to control for some of these measures, and to perform further inter-subject comparisons. In our study, three out of eleven participants were at the age of 60–65. These patients did not show any irregularities in their pattern of performance, compared to younger participants–they all demonstrated a clear improvement through sessions and sequences. Nevertheless, future research should aspire to maintain a relatively homogeneous sample and control for demographic parameters (such as age, gender, education etc.) and clinical parameters (such as time from injury, type and severity of injury etc.).

Once we are able to conduct this study with a larger sample, several inter-group comparisons should be made. First, randomly assigning patients to sequence A or B as the observed sequence, will allow us to exclude the possibility of sequence-specific effects (as performed with healthy participants, by Korman et al., 2007).

Other paradigms should be conducted to explore the possibility of order-specific effects, in order to rule out cognitive fatigue as an alternative explanation for lower performance in sequence B. To do so, one cannot simply counterbalance the order of the observed and unobserved sequence, due to the possibility of an interference effect of the motor-trained sequence vs. the observed sequence (Balas et al., 2007). Thus, an alternative protocol may be conducted, in which patients undergo similar observational training for two different sequences, while counterbalancing for order (in presentation as well as in testing). Equal performance of both sequences may corroborate the notion that the effect found in the current study is due to skill observation rather than order of exposure.

Due to the exploratory nature of this study, our protocol was rather simple and was designed as a series of within-subject tests–all participants suffered a moderate-severe TBI injury, and identically performed all stages of the research. After establishing the notion that these patients can benefit from a single and relatively basic experience of observational learning, future research should include an elaboration of our research model, with several control groups. For example, using healthy control participants, may enable us to evaluate specific characterization of observational learning in healthy subjects compared to TBI patients.

Also, as mentioned earlier, the testing protocol, which intrinsically included multiple physical repetitions (indirectly) served as a motor practice for both sequences. Although the gap between sequence A and B had remained identical in the retention test 2 weeks later, it is not clear which element of learning (the physical or observational) had a greater effect with patients by the end of the study. Previous work in the field of observational skill-learning suggested that practice by observation, although highly effective, cannot measure up to actual physical practice (Hesseg et al., 2016; Hesseg, 2017). Additional studies claimed that a combination of observation and of physical practice is the superior and preferable form of skill learning for both healthy participants and Cerebro Vascular Attack (CVA) patients (Blandin et al., 1999). Thus, elaborating our model to compare “pure” observational learning vs. mixed observational-physical practice or groups of physical practice alone, could deepen our understanding on the relative contribution of observational vs. physical cues to procedural learning with TBI patients.

## Conclusion

The current study was designed to assess the potential of TBI patients to profit from a novel method of skill learning. Data revealed that patients benefited greatly from observational training, while some patients gained more than others, especially those with moderate FIM scores. This form of observational training may have several advantages for these patients, who often suffer from severe limitations in motor function. First, it may contribute to reorganization processes and neuro-plasticity of the motor system. Second, observing a desired movement before engaging any actual motor-training, may encourage the replacement of pathological patterns with a corrected representation of that movement, as shown in previous studies (Bobath, 1990). These theories invite further investigation, not only to ensure and replicate our results across different timeframes and patient populations, but also to better understand the nature of observational skill-learning and how it should be assimilated with TBI patients.

## Data availability statement

The raw data supporting the conclusions of this article will be made available by the authors, without undue reservation.

## Ethics statement

The studies involving human participants were reviewed and approved by the Loewenstein Rehabilitation Medical Center IRB. The patients or their legal guardians provided their written informed consent to participate in this study.

## Author contributions

EA and YS performed the planning and initiation of the experiment. RM-H, AK, YS, and EA designed the research protocol. EA and YS performed the execution of the experiment, data analysis, and writing of the manuscript, with the consulting and assistance of RD, RM-H, and AK. All authors contributed to the article and approved the submitted version.
